# Population pharmacokinetics and electrocardiographic effects of dihydroartemisinin–piperaquine in healthy volunteers

**DOI:** 10.1111/bcp.13372

**Published:** 2017-08-16

**Authors:** Palang Chotsiri, Thanaporn Wattanakul, Richard M. Hoglund, Borimas Hanboonkunupakarn, Sasithon Pukrittayakamee, Daniel Blessborn, Podjanee Jittamala, Nicholas J. White, Nicholas P.J. Day, Joel Tarning

**Affiliations:** ^1^ Mahidol‐Oxford Tropical Medicine Research Unit Mahidol University Bangkok Thailand; ^2^ Faculty of Tropical Medicine Mahidol University Bangkok Thailand; ^3^ Centre for Tropical Medicine and Global Health, Nuffield Department of Clinical Medicine University of Oxford Oxford UK

**Keywords:** dihydroartemisinin, interaction, piperaquine, population pharmacokinetic–pharmacodynamic model, primaquine, QT prolongation

## Abstract

**Aims:**

The aims of the present study were to evaluate the pharmacokinetic properties of dihydroartemisinin (DHA) and piperaquine, potential drug–drug interactions with concomitant primaquine treatment, and piperaquine effects on the electrocardiogram in healthy volunteers.

**Methods:**

The population pharmacokinetic properties of DHA and piperaquine were assessed in 16 healthy Thai adults using an open‐label, randomized, crossover study. Drug concentration–time data and electrocardiographic measurements were evaluated with nonlinear mixed‐effects modelling.

**Results:**

The developed models described DHA and piperaquine population pharmacokinetics accurately. Concomitant treatment with primaquine did not affect the pharmacokinetic properties of DHA or piperaquine. A linear pharmacokinetic–pharmacodynamic model described satisfactorily the relationship between the individually corrected QT intervals and piperaquine concentrations; the population mean QT interval increased by 4.17 ms per 100 ng ml^–1^ increase in piperaquine plasma concentration. Simulations from the final model showed that monthly and bimonthly mass drug administration in healthy subjects would result in median maximum QT interval prolongations of 18.9 ms and 16.8 ms, respectively, and would be very unlikely to result in prolongation of more than 50 ms. A single low dose of primaquine can be added safely to the existing DHA–piperaquine treatment in areas of multiresistant Plasmodium falciparum malaria.

**Conclusions:**

Pharmacokinetic–pharmacodynamic modelling and simulation in healthy adult volunteers suggested that therapeutic doses of DHA–piperaquine in the prevention or treatment of P. falciparum malaria are unlikely to be associated with dangerous QT prolongation.

## What is Already Known about this Subject

- Dihydroartemisinin (DHA)–piperaquine has shown excellent efficacy and tolerability in the treatment of malaria. However, concerns have been raised about potentially harmful cardiotoxic effects associated with piperaquine administration.
- The World Health Organization has recently suggested adding a single low dose of primaquine, a drug with rapid gametocytocidal activity against *Plasmodium falciparum*, to reduce transmissibility in areas of low malaria transmission.

## What this Study Adds

- This was the first population pharmacokinetic and electrocardiographic safety study of DHA–piperaquine, with and without primaquine coadministration.
- No drug–drug interactions between DHA–piperaquine and primaquine were identified using population pharmacokinetic modelling. The developed model describing piperaquine‐associated QT prolongations indicated that therapeutic concentrations of piperaquine are safe.
- The pharmacokinetic modelling conducted here demonstrated that a single low dose of primaquine can be added safely to the existing DHA–piperaquine treatment in areas of multiresistant *P. falciparum* malaria. Pharmacokinetic–pharmacodynamic modelling and simulation suggested that therapeutic doses of DHA–piperaquine in prevention or treatment of malaria are unlikely to be associated with dangerous QT prolongation.

## Introduction

Dihydroartemisinin (DHA)–piperaquine is currently one of five artemisinin‐based combination therapies (ACTs) recommended by the World Health Organization (WHO) for the treatment of *Plasmodium falciparum* malaria 1, 2, 3. It has also proved to be well tolerated and effective in mass treatments and intermittent preventive therapies 4, 5. DHA is a potent antimalarial compound but it is rapidly eliminated from the systemic circulation (elimination half‐life 1–2 h) 6, 7, 8. By contrast, piperaquine has a large apparent volume of distribution and a long terminal elimination half‐life (20–30 days). Thus, in the DHA‐piperaquine ACT, the slowly eliminated piperaquine removes those parasites remaining after the 3‐day course of DHA 9. Artemisinin resistance in *P. falciparum* has emerged in South‐East Asia 10, 11, threatening current elimination efforts and leading to partner drug resistance. Mass drug administration with DHA–piperaquine is one approach to resistance containment but proposed extensive use in healthy people emphasizes the need to assess potential cardiovascular toxicity risks 12, 13.

Primaquine is the only available drug for the radical cure of Plasmodium vivax malaria. A single low dose of primaquine is also recommended by the WHO as a gametocytocide in acute *P. falciparum* malaria 4. This single 0.25 mg base kg^–1^ dose is considered unlikely to cause serious toxicity in patients with glucose‐6‐phosphate dehydrogenase deficiency, so it should be given to all nonpregnant patients above 6 months of age with *P. falciparum* malaria in low transmission settings 14.

The potential for high doses of quinoline‐related compounds to cause cardiovascular toxicity has been recognized since the first introduction of the cinchona alkaloids. Quinidine, the diastereomer of quinine, is the prototype for medicines causing delayed ventricular repolarization, which is manifest as marked QT prolongation (once termed the ‘quinidine effect’) on the electrocardiogram (ECG). This results in both antiarrhythmic and proarrhythmic effects. QT prolongation may be associated with potentially lethal polymorphic ventricular tachycardia (i.e. torsades de pointes), particularly in patients with congenitally long QT intervals or those with other predisposing factors. The most extreme effects caused by antimalarial drugs occurred with halofantrine, which was clearly associated with sudden death 15. Although QT prolongation is associated with several structurally related antimalarial agents, halofantrine is the only compound that has been associated with sudden unexplained death. Piperaquine is structurally similar to chloroquine, which also causes consistent QT prolongation 16. Concerns have been raised regarding the potential for DHA–piperaquine to cause cardiotoxicity. Several studies have reported a significant QT prolongation associated with DHA–piperaquine treatment 17, 18, 19. A recent study of a high piperaquine dose (50% increased dosage compared with standard treatment) in Cambodian soldiers reported a substantial prolongation of the Fridericia‐corrected QT (QTcF) interval 20 and the study was halted because of cardiovascular safety concerns, although the machine read the QU rather than the QT intervals. A study in Cambodian children and adults with uncomplicated *P. falciparum* malaria showed a small but significant prolongation of the Bazett‐corrected median QT (QTcB) interval of 11 [95% confidence interval (CI) 4, 18] ms after receiving a standard age‐based dosage of DHA–piperaquine 21. A large multicentre, prospective, observational study in African patients receiving a standard 3‐day treatment of DHA–piperaquine showed that only three out of 1002 evaluated patients had a QTcF interval above 500 ms and less than 10% of patients had a maximum QTcF prolongation above 60 ms 22. The interpretation of electrocardiographic changes during the treatment of malaria is confounded by systematic changes that occur during recovery and result in QT lengthening, so drug effects are better assessed in healthy subjects, who are also more representative of populations receiving mass treatments.

The present study aimed to investigate the population pharmacokinetic properties of DHA and piperaquine, identify potential drug–drug interactions with primaquine and quantify the relationship between piperaquine exposure and QT prolongation in healthy volunteers using a nonlinear mixed‐effects modelling approach.

## Materials and methods

### Study design

The study was conducted at the Faculty of Tropical Medicine, Mahidol University, Bangkok, Thailand. The clinical details and noncompartmental pharmacokinetic results of the study have been reported in full elsewhere 23. Study approval was obtained by the ethics committee of the Faculty of Tropical Medicine, Mahidol University, Bangkok, Thailand (reference number TMEC 12‐004, approval number MUTM 2012‐009‐01), and by the Oxford University Tropical Research Ethics Committee (OXTREC 58‐11). The study was registered at Clinicaltrials.gov (NCT01525511, 16 January 2012). The methods used were in accordance with the approved guidelines.

The study aims were explained in full to the volunteers, and written informed consent was obtained from all subjects before their participation. At admission, a full medical history was taken, a physical examination and complete blood count were carried out and blood glucose levels were measured. Participants with malaria or with glucose‐6‐phosphate dehydrogenase (G6PD) deficiency, pregnant women and lactating women were excluded from the study. Safety was analysed based on adverse events, physical examination, vital signs, clinical laboratory parameters, 12‐lead ECG and methaemoglobin levels.

The study had an open‐label, randomized, three‐way, crossover design and was conducted in 16 healthy Thai volunteers. It was a descriptive pharmacokinetic–pharmacodynamic study and no formal sample size calculations were performed. However, 16 subjects were chosen on the basis of the observed variability in the pharmacokinetic parameters of the study drugs, and therefore assumed to generate a reasonable degree of accuracy in parameter estimates. All volunteers received primaquine alone in the first phase, followed by a washout period of 1 week. In the second and third phases, volunteers received DHA–piperaquine alone and DHA–piperaquine coadministered with primaquine at random, with an intervening washout period of 8 weeks. Study drug regimens comprised two tablets of primaquine (each tablet containing 15 mg primaquine base) and three tablets of co‐formulated DHA–piperaquine (each tablet containing 40 mg DHA and 320 mg piperaquine phosphate). Study drugs were administered in the morning, 30 min after a light meal (~200 kcal and 8 g fat) with a glass of water.

Subjects were rested for at least 20 min before ECG measurements were taken (ECG‐1250 K, Nihon Kohden, Japan). 12‐lead ECG measurements were performed twice before drug administration, and at 1, 2, 4, 8, 12, and 24 h after each study drug administration. The ECGs were recorded at 10 mm mV^–1^ sensitivity, and 25 mm s^–1^ paper speed. Automatic readouts of all ECG measurements were collected but all ECGs with a reported QT interval greater than 450 ms were manually adjudicated by a research physician (unblinded) and a cardiologist (blinded). Other abnormal ECG waveforms were read by a cardiologist. Observed QT intervals were later corrected for heart rate by both the Fridericia and Bazett formulae 24. Data‐driven individual and study population correction factors were also evaluated (see section on methodology, below).

Venous blood samples (2 ml) were collected into fluoride–oxalate blood collection tubes. Blood samples were taken at 0 (predose), 0.25, 0.5, 1, 1.5, 2, 3, 4, 6, 8, 10, 12, and 24 h postdose. Additional blood samples were taken at days 3, 4, 7, 11, 15, 22, and 36 for piperaquine drug measurements. The exact drug administration and sampling times were recorded. Blood and plasma were separated by centrifugation at 2000 × *g* at 4°C for 7 min. All plasma samples were stored below −70°C and transferred on dry ice to the Department of Clinical Pharmacology, Mahidol‐Oxford Tropical Medicine Research Unit (MORU), Bangkok, Thailand, for drug quantification.

### Drug quantification

Plasma concentrations of DHA and piperaquine were measured using solid‐phase extraction followed by liquid chromatography coupled with tandem mass spectrometry 25, 26. Quality control samples at low, middle and high concentration (5.87, 117 and 1880 ng ml^–1^ for DHA and 4.50, 20.0 and 400 ng ml^–1^ for piperaquine) were analysed in triplicate within each batch of study samples, to ensure the accuracy and precision of the drug assay. The relative standard deviations (% CV) were 3.49%, 2.54% and 1.87% for the DHA quality control samples and 4.76%, 2.60% and, 2.82% for the piperaquine quality control samples. The lower limit of quantification (LLOQ) was set to be 2.00 ng ml^–1^ for DHA and 1.50 ng ml^–1^ for piperaquine. The laboratory is a participant in the QA/QC proficiency testing programme supported by the Worldwide Antimalarial Resistance Network 27.

### Population pharmacokinetic analysis

DHA and piperaquine plasma concentrations were transformed into their natural logarithms and analysed using a nonlinear mixed‐effects modelling approach in NONMEM version 7.3 (Icon Development Solution, Ellicott City, MD, USA). Pirana version 2.9.0 28, Perl‐speaks‐NONMEM version 3.5.3 (PsN) 29 and Xpose version 4.0 30 were used for automation, model evaluation and diagnostics during the model‐building process. The first‐order conditional estimation method with interactions (continuous data only) or the Laplacian estimation method (a combination of continuous and categorical data) was used throughout modelling and simulation. Piperaquine concentrations below the LLOQ were omitted as only 2.3% of the samples were measured to be below this level. However, a relatively large fraction of DHA concentrations were below the LLOQ (15% of all data, and 7.0% of data in the elimination phase). Therefore, two LLOQ methods were evaluated during the model‐building process 31. Data below the LLOQ were omitted (M1 method) or modelled as categorical data (M3 method). Model fitness was evaluated primarily by the objective function value (OFV; calculated by NONMEM as proportional to −2 × log‐likelihood of the data). Model discrimination between two hierarchical models was determined by a likelihood ratio test, based on the chi‐square distribution of the OFV (i.e. *P*‐value <0.05 then ΔOFV >3.84, at 1 degree of freedom difference).

One‐, two‐, three‐ and four‐compartment structural disposition models were evaluated for DHA and piperaquine. The best performing model was used to evaluate the absorption characteristics of DHA and piperaquine (i.e. first‐order absorption with and without lag time, zero‐order absorption and transit absorption). The transit compartment absorption model is a more mechanistic description of delayed absorption compared with the dichotomous properties of a lag‐time model 32.

Pharmacokinetic parameters were assumed to be log‐normally distributed and therefore implemented as an exponential between‐subject variability, as in Equation (1).

(1) $$\theta_{i}=\theta \;\cdot \;e^{\eta_{i,\theta }}$$

where θ_i_ is individual *i*’s parameter estimate, θ is the typical parameter estimate of the population and η_i,θ_ is the between‐subject variability for individual *i*, which is normal distributed with a zero mean and variance ω^2^. The between‐occasion variability (the variability between administration of the study doses) was also investigated, as in Equation (2):

(2) $$\theta_{\mathrm{ij}}=\theta \;\cdot \;e^{\eta_{i,\theta }+\kappa_{j,\theta }}$$

where κ_j,θ_ is the between‐occasion variability of the pharmacokinetic parameter θ at the *j*th dosing occasion. Between‐subject and between‐occasion variability was also evaluated on the relative bioavailability, fixed to unity for the population, to allow for the observed high variability in the absorption of the study drugs. Estimated between‐subject and between‐occasion variability below 10% or when estimated with poor precision (RSE > 50%) were fixed to zero. Residual unexplained variability was modelled as an additive error on the log‐transformed observed concentrations (equivalent to an exponential error on an arithmetic scale).

Body weight was introduced into the pharmacokinetic model as a fixed allometric function on all volume, clearance and distribution parameters, centred on the median body weight (64 kg) of the study population, as in Equation (3) and (4) 33:

(3) $${\mathrm{CL}}_{i}=\mathrm{CL}\times e^{\eta_{i,\theta }}\times {\left(\frac{{\mathrm{BW}}_{i}}{64}\right)}^{0.75}$$

(4) $$V_{i}=V\times e^{\eta_{i,\theta }}\times {\left(\frac{{\mathrm{BW}}_{i}}{64}\right)}^{1.00}$$

where CL_*i*_ represents the individual clearance value, CL represents the typical population value of clearance, BW_*i*_ represents the individual body weight, V_*i*_ represents the individual volume of distribution, and V represents the typical population value of volume of distribution.

All continuous and categorical covariates (aspartate aminotransferase, alanine aminotransferase, alkaline phosphatase, haemoglobin, blood urea nitrogen, serum creatinine level, albumin level, primaquine coadministration and age) were investigated by using a stepwise forward inclusion (*P*‐value <0.05), followed by stepwise backward elimination (*P*‐value >0.001). A strict *P*‐value of 0.001 for the backward elimination was used as there were relatively few subjects in the present study 34. Gender was not evaluated as a covariate owing to the substantial imbalance between male and female subjects (five males out of 16 subjects). The effect of primaquine coadministration was also modelled separately, using a full covariate approach in which the primaquine coadministration was implemented as a categorical covariate on all pharmacokinetic parameters (except relative bioavailability owing to identifiability issues) in the final pharmacokinetic model. The full covariate models were bootstrapped (*n* = 1000) to determine a potentially influential drug–drug interaction on primary and secondary pharmacokinetic parameters. A primaquine‐dependent change of more than ±25% in parameter estimates was deemed a clinically relevant drug–drug interaction.

Potential model misspecification and systematic errors were evaluated by basic goodness‐of‐fit diagnostics. Eta and epsilon shrinkages were used to assess the ability to detect model misspecifications in goodness‐of‐fit diagnostics 35. Model robustness and nonparametric confidence intervals were evaluated by bootstrap diagnostics (*n* = 1000). Predictive performances of the final models were illustrated by prediction‐corrected visual and numerical predictive checks (*n* = 2000) 36. The 5th, 50th and 95th percentiles of the observed concentrations were overlaid with the 95% CIs of each simulated percentile, to detect model bias.

### Population cardiac electrophysiological pharmacodynamics of piperaquine

The observed QT interval must be corrected for heart rate in order to compare QT intervals between and within patients. Observed QT measurements were corrected by the traditionally used Bazett and Fridericia formulae (i.e. fixing the exponent (α) to 1/2 and 1/3, respectively, in Equation (5)). Furthermore, all observed individual QT and RR intervals from the placebo arm (i.e. primaquine‐alone arm) in a subject were used to determine the optimal individual QT correction factor (α) for each subject using ordinary least‐squares fit (Equation (5)). The calculated individual correction factor for a particular subject was then applied to all measured QT intervals for that subject, in order to generate corrected QT intervals (QTc) 24.

(5) $$\mathrm{QT}={\mathrm{QT}}_{C}\times {\mathrm{RR}}^{\alpha }$$

The appropriateness of the applied correction methods was evaluated by individual linear regression analysis of QTc *vs*. RR. The relationship between piperaquine drug concentrations and QRS, JT (i.e. QT – QRS) and QT intervals was evaluated with ordinary linear regression to assess the most appropriate modelling approach. The individually corrected QT interval was deemed the most appropriate measurement (see results section, below) in this particular analysis and therefore carried forward throughout modelling and simulation.

QTc prolongations (ΔQTc) were calculated by subtracting the baseline QTc interval (QTc_Baseline_) from the observed QTc intervals after study drug administration (QTc_Post‐dose_), as in Equation (6). Double‐delta corrections are commonly performed to adjust for the observed circadian rhythm of ECG measurements 37. Thus, double‐delta‐corrected QTc prolongations (ΔΔQTc) were calculated by subtracting the placebo arm ΔQTc from the treatment arm ΔQTc, as in Equation (7). The primaquine‐alone arm was used as the placebo arm. Although primaquine can be shown in experimental conditions to affect ion channels, and notably to block the human ether‐à‐go‐go‐related gene (hERG) potassium channel, the active concentrations are substantially higher than those likely to occur in humans taking low oral doses 38, 39. Furthermore, there was no correlation between ΔQTc *vs*. primaquine concentrations in the primaquine alone arm when evaluated using ordinary linear regression (i.e. the slope did not deviate significantly from zero).

(6) $$\text{ΔQTc}={\mathrm{QTc}}_{\text{Post}‐\text{dose}}-{\mathrm{QTc}}_{\text{Baseline}}$$

(7) $$\Delta \Delta \text{QTc}={\left[\text{ΔQTc}\right]}_{\text{Treatment}}-{\left[\text{ΔQTc}\right]}_{\text{Placebo}}$$

Calculated ΔΔQTc‐prolongations were used as the pharmacodynamic endpoint. Individually predicted piperaquine concentrations (C_P_) were obtained from imputing individual pharmacokinetic parameter estimates directly into the pharmacodynamic model. The relationship between drug exposure and QT prolongation was evaluated initially by a linear direct‐response pharmacodynamic model, as in Equation (8).

(8) $$\Delta \Delta \mathrm{QTc}=\left(\theta_{1}+\eta_{1}\right)+\theta_{2}\times C_{P}\left(t\right)+\varepsilon_{i}$$

where θ_1_ represents the typical baseline ΔΔQTc prolongation, η_1_ is the normally distributed between‐subject variability, θ_2_ is the slope of the exposure–response relationship and ε_i_ is the normally distributed residual error. Different exposure–response relationships (i.e. power model and E_MAX_ model) were also investigated during the model development process. Hysteresis was investigated to account for a possible delayed exposure–response relationship (i.e. turn‐over and link models).

Age, gender, electrolyte levels (i.e. potassium and sodium) at admission and concomitant primaquine administration were evaluated as linear covariates on the piperaquine‐related ΔΔQTc prolongation, using a stepwise addition–deletion approach (as described above). Model evaluation and diagnostics were performed in the same manner as for the pharmacokinetic modelling approach.

The final population pharmacokinetic–pharmacodynamic model was used to simulate QTc prolongation at different piperaquine concentrations. Single piperaquine doses ranging from 100 mg to 2000 mg were simulated (a total of 20 000 simulated subjects) in order to cover a wide range of possible piperaquine concentrations. Simulated piperaquine concentrations and the associated ΔΔQTc prolongation were overlaid with the observed data to determine the piperaquine concentrations resulting in predicted clinically important QT prolongations (> 60 ms). The final pharmacokinetic–pharmacodynamic model of piperaquine was also used to simulate expected QT prolongation in mass drug administration scenarios. A total of 1000 healthy subjects (body weight of 60 kg), receiving a standard 3‐day treatment regimen every 4 weeks or every 8 weeks for a total duration of 1 year, were simulated. The maximum QT prolongation, in each simulated subject, after each round of drug administration was visualized in order to characterize the likely effects of DHA–piperaquine in malaria elimination campaigns. A total of 1000 hypothetical patients (body weight of 60 kg), receiving a standard 3‐day treatment regimen of DHA–piperaquine 4, were also simulated based on a population pharmacokinetic model in nonpregnant women with uncomplicated *P. falciparum* malaria to evaluate the expected QT prolongation in a patient population 8.

### Nomenclature of targets and ligands

Key protein targets and ligands in this article are hyperlinked to corresponding entries in http://www.guidetopharmacology.org, the common portal for data from the IUPHAR/BPS Guide to PHARMACOLOGY 40, and are permanently archived in the Concise Guide to PHARMACOLOGY 2015/16 41.

## Results

The frequent sampling and the crossover design produced ideal data for pharmacokinetic–pharmacodynamic modelling. All 16 volunteers completed the study protocol, and tolerated the treatments well, with no reported serious adverse events. The study was conducted between 18 June 2012 and 2 November 2012. The clinical safety results have been published in full elsewhere 23. The full demographic characteristics are presented in Table 1.

**Table 1:** Subject baseline demographics and covariates

	Median (range)	Mean ± SD
**Body weight (kg)**	64.1 (54.0–71.4)	62.7 ± 5.89
**Body height (cm)**	165 (154–175)	165 ± 6.06
**Age (years)**	40 (22–53)	37.4 ± 9.22
**Heart rate (per min)**	76.0 (64.0–90.0)	74.6 ± 6.96
**Body temperature (°C)**	36.8 (36.3–37.2)	36.8 ± 0.250
**Systolic blood pressure (mmHg)**	110 (100–120)	110 ± 6.85
**Diastolic blood pressure (mmHg)**	60.0 (50.0–80.0)	64.4 ± 8.92
**Ventricular rate (beats per min)**	68.0 (51.0–90.0)	67.4 ± 9.67
**PR interval (ms)**	161 (128–200)	161 ± 20.5
**QRS duration (ms)**	91.0 (78.0–102)	88.9 ± 7.00
**Uncorrected QT interval (ms)**	395 (370–446)	400 ± 22.1
**QTc interval (ms)**	422 (386–466)	422 ± 18.5
**Glucose (mg dl** ^**–1**^ **)**	85.0 (75.0–115)	87.9 ± 9.95
**Albumin (g dl** ^**–1**^ **)**	4.25 (3.90–5.00)	4.29 ± 0.305
**Alkaline phosphatase (U l** ^**–1**^ **)**	51.0 (30.0–71.0)	52.6 ± 11.6
**Aspartate aminotransferase (U l** ^**–1**^ **)**	16.5 (11.0–21.0)	16.3 ± 3.05
**Alanine aminotransferase (U l** ^**–1**^ **)**	14.5 (7.00–30.0)	16.1 ± 6.87
**Sodium (mmol l** ^**–1**^ **)**	140 (135–146)	140 ± 3.01
**Potassium (mmol l** ^**–1**^ **)**	4.25 (3.50–4.80)	4.18 ± 0.390

SD, standard deviation

### Population pharmacokinetic properties of DHA

A total of 384 DHA plasma samples were collected. A two‐compartment disposition model proved superior to a one‐compartment model, both when omitting concentrations measured below the LLOQ (ΔOFV = −26.0) and when implementing them as categorical data using the M3 method (ΔOFV = −12.3). This confirmed that the improved model fit was not because of data censoring. Adding an extra third disposition compartment resulted in a minor improvement in model fit (ΔOFV = −6.55; *P* > 0.01). In addition, the terminal half‐life estimated from the three‐compartment model was somewhat long (median half‐life of 3.11 h) compared with previous reports (0.145–2.5 h) 7, 8. Therefore, the two‐compartment disposition model was carried forward. Omitting concentrations below the LLOQ did not show any model misspecification in the fraction of censored observations and resulted in similar model performance to that using the M3 method. The approach of omitting concentrations below the LLOQ was therefore deemed appropriate.

A transit compartment absorption model with six transit compartments was superior to all other absorption models evaluated (ΔOFV > −258). Estimating both the transit rate between transit compartments and the absorption rate from the last transit compartment to the central compartment resulted in a significantly improved model fit compared with when setting them to be equal (ΔOFV = −17.6).

Implementing body weight as a fixed allometric function on all clearance and volume parameters did not improve the model fit (ΔOFV = 0.819). However, it was retained in the final model based on the strong biological prior and previously published results 7. No significant covariates were identified in the stepwise covariate approach. The observed data showed substantial between‐occasion variability in the absorption of DHA, with additional between‐subject variability in the elimination clearance of DHA.

**Figure 1:**
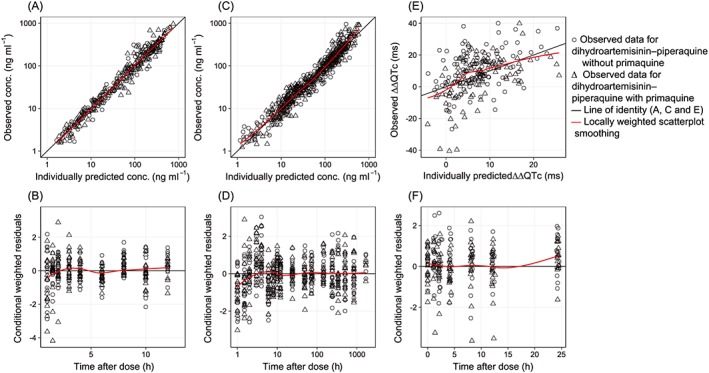
Goodness‐of‐fit plots of the final population pharmacokinetic models of dihydroartemisinin (A, B) and piperaquine (C, D), and the population pharmacokinetic–pharmacodynamic model describing the double‐delta‐corrected QTc prolongation (ΔΔQTc) interval (E, F). conc., concentration

**Figure 2:**
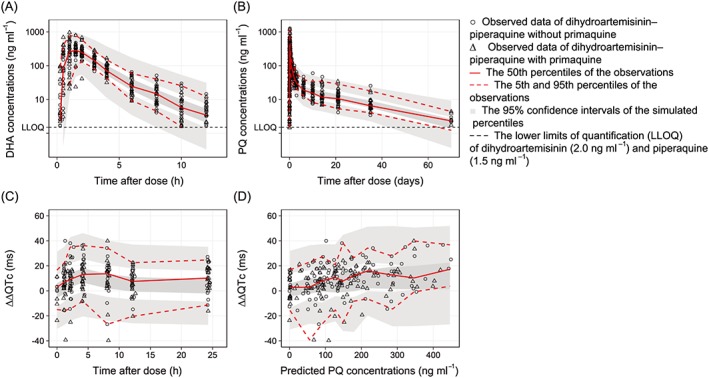
Visual predictive plots of the final population pharmacokinetic–pharmacodynamic models of dihydroartemisinin (DHA) *vs*. time (A), piperaquine (PQ) *vs*. time (B), the double‐delta‐corrected QTc prolongation (ΔΔQTc) *vs*. time (C), and ΔΔQTc *vs*. predicted piperaquine concentrations (D)

The final model showed a satisfactory goodness of fit (Figure 1) and predictive performance, as illustrated by the visual predictive check (Figure 2A). Eta and epsilon shrinkages were generally low (<20%) except for the absorption rate constant (37.6% and 23.7% shrinkage on study occasions 1 and 2, respectively). A numerical predictive check (*n* = 2000) resulted in 1.84% (95% CI 1.23%, 10.4%) and 3.99% (95% CI 1.53%, 10.1%) of DHA observations below and above, respectively, the simulated 90% prediction interval. Pharmacokinetic parameter estimates from the final model and corresponding secondary parameters are summarized in Table 2 and Table 3, respectively.

**Table 2:** Parameter estimates from the final population pharmacokinetic–pharmacodynamic model of dihydroartemisinin and piperaquine in healthy volunteers

	Population estimates^c^ (%RSE^d^)	95% CI^d^	%CV^c^ of BSV/BOV* (%RSE^d^)	95% CI^d^
***Pharmacokinetic parameters of dihydroartemisinin***				
**F (%)**	100 Fixed		35.9% (20.1%)*	21.4%–50.4%
**MTT (h)**	0.567 (11.4%)	0.527–0.818	52.6% (14.2%)*	36.0%–67.6%
**k**_**a**_ **(h** ^**−1**^ **)**	2.89 (37.1%)	1.88–6.99	89.0% (23.7%)*	46.0%–169%
**CL/F (l h** ^**−1**^ **)**	148 (10.6%)	121–183	23.1% (14.2%)	15.2%–27.6%
**V_C_/F (l)**	214 (16.9%)	148–287		
**Q**_**P**_ **/F (l h** ^**−1**^ **)**	28.5 (26.0%)	15.5–44.1		
**V_P_/F (l)**	65.9 (19.1%)	42.5–91.5		
**σ_PK_**	0.358 (9.07%)	0.292–0.418		
***Pharmacokinetic parameters of piperaquine***				
**F (%)**	100 Fixed		17.9% (34.0%)	0.178%–26.1%
			19.1% (13.3%)*	13.5%–23.3%
**MTT (h)**	3.13 (9.42%)	2.66–3.84	32.2% (13.4%)*	21.1%–37.8%
**CL/F (l h** ^**−1**^ **)**	27.4 (5.50%)	24.6–30.4	10.9% (37.2%)	0.109%–15.72%
**V_C_/F (l)**	751 (23.5%)	470–1160	42.4% (40.9%)	0.406%–62.9%
**Q**_**P1**_ **/F (l h** ^**−1**^ **)**	206 (9.56%)	166–242		
**V_P1_/F (l)**	1900 (8.23%)	1660–2260		
**Q**_**P2**_ **/F (l h** ^**−1**^ **)**	71.5 (9.01%)	58.5–84.4	24.1% (36.3%)	0.203%–37.3%
**V_P2_/F (l)**	13 500 (8.95%)	11 400–16 000		
**σ_PK_**	0.137 (9.22%)	0.111–0.161		
***Pharmacodynamic parameters***				
**BASE (ms)**	0 Fixed		15.9 (33.4%)	0.973–43.11
**SLOPE [ms (ng ml** ^**−1**^ **)** ^**−1**^ **]**	0.0417 (12.5%)	0.0313–0.0511		
**σ_PD_ (ms)**	146 (25.5%)	82.1–220		

ΔΔQTc, double‐delta‐corrected QTc prolongation; BASE, baseline; BOV, between‐occasion variability; BSV, between‐subject variability; CI, confidence interval; CL/F, oral clearance; %CV, coefficient of variation; F, relative bioavailability; k_a_, absorption rate constant from last transit compartment to central compartment; MTT, mean transit time; Q_P_/F, inter‐compartment clearance; σ_PK_, residual exponential error variance of drug measurements; σ_PD_, residual additive error variance of ΔΔQTc prolongation; %RSE, relative standard deviation; SLOPE, slope parameter of the relationship between piperaquine concentration and ΔΔQTc‐prolongation; V_C_/F, apparent central volume of distribution; V_P_/F, apparent peripheral volume of distribution.

* Between‐occasion variability

c Computed population mean parameter estimates from NONMEM. Parameter estimates are based on the typical individual in the population with a body weight of 64 kg. BSV and BOV are presented as the %CV, calculated as $100\times \sqrt{\exp\left(\text{estimate}\right)-1}$

d Based on nonparametric bootstrap diagnostics (*n* = 1000 samples). Parameter precision is presented as %RSE, calculated as $100\times \frac{\text{standard deviation}}{\text{mean value}}$. The 95% CI is calculated as the 2.5th to 97.5th percentile of bootstrap estimates

**Table 3:** Secondary parameter estimates of dihydroartemisinin and piperaquine in healthy volunteers with and without primaquine coadministration

	With primaquine	Without primaquine	*P*‐value^b^
***Dihydroartemisinin*** a			
**C**_**MAX**_ **(ng ml** ^**–1**^ **)**	357 (252–417)	361 (247–414)	0.910
**T**_**MAX**_ **(h)**	1.27 (0.988–1.50)	1.30 (1.04–1.51)	0.652
**t**_**1/2**_ **(h)**	2.20 (1.99–2.35)	2.20 (1.99–2.35)	N/A^c^
**AUC (h × ng ml** ^**–1**^ **)**	798 (575–1154)	767 (690–1102)	0.597
***Piperaquine*** a			
**C**_**MAX**_ **(ng ml** ^**–1**^ **)**	300 (128–593)	332 (157–544)	0.706
**T**_**MAX**_ **(h)**	3.98 (2.30–6.71)	3.76 (3.12–5.45)	0.597
**t**_**1/2**_ **(days)**	22.1 (20.8–23.4)	22.1 (20.8–23.4)	NA^c^
**AUC (h × ng ml** ^**–1**^ **)**	17 700 (13800–30 800)	19 600 (10500–33 200)	0.980
**Day 7 conc. (ng ml** ^**–1**^ **)**	16.7 (13.8–28.9)	18.3 (10.5–34.9)	0.980

AUC; area under the concentration–time curve from time zero to infinity; C_MAX_, maximum concentration; Day 7 conc., day 7 concentration of piperaquine; NA, not available; T_MAX_, time to maximum concentrations; t_1/2_, terminal half‐life

a Median parameter estimates (range) were obtained from the Bayesian *post hoc* estimates of the final population pharmacokinetic models

b *P*‐values were calculated using the Wilcoxon matched‐pairs signed rank test

t_1/2_ value estimated from the model were identical between the two groups

### Population pharmacokinetic properties of piperaquine

A total of 623 piperaquine plasma samples were collected in the study. A three‐compartment disposition model resulted in a significantly improved model fit compared with a two‐compartment disposition model (ΔOFV = −297). No further improvement was seen with an additional disposition compartment (ΔOFV = −0.500). A transit compartment absorption model with two transit compartments was superior to all other models evaluated (ΔOFV > −452). There was no significant change in model fit when the transit rate between transit compartments and the absorption rate from the last transit compartment to the central compartment were set to be equal (ΔOFV = 0.564).

Implementing body weight as a fixed allometric function on all clearance and volume parameters resulted in an improved model fit (ΔOFV = −5.95). No other covariates were significant in the stepwise covariate approach. The observed data showed substantial between‐subject and between‐occasion variability in the absorption of piperaquine, with additional between‐subject variability in the elimination clearance, the inter‐compartmental clearance and the central volume of distribution of piperaquine.

The final model showed a satisfactory goodness of fit (Figure 1) and predictive performance, as illustrated by the visual predictive check (Figure 2B). Moderate eta and epsilon shrinkages were seen in the final model (i.e. between 20% and 30%) except for clearance, which showed a somewhat higher shrinkage of 35.3%. A numerical predictive check (*n* = 2000) resulted in 3.79% (95% CI 2.14%, 8.73%) and 4.12% (95% CI 2.31%, 8.73%) of piperaquine observations below and above, respectively, the simulated 90% prediction interval. Pharmacokinetic parameter estimates from the final model and corresponding secondary parameters are summarized in Table 2 and Table 3, respectively.

### Drug–drug interactions

**Figure 3:**
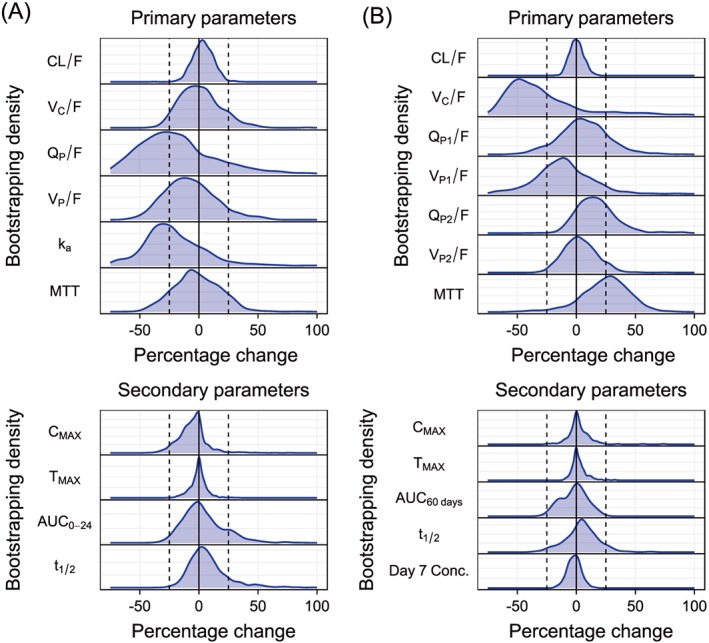
Effect of primaquine coadministration on the pharmacokinetic parameters of dihydroartemisinin (A) and piperaquine (B) when using a full covariate approach. The top panels illustrate primary pharmacokinetic parameters and the lower panels illustrate secondary derived pharmacokinetic parameters. The y‐axes represent the density of parameter estimates from 1000 bootstraps. The vertical dashed lines represent a covariate effect of ±25%, assumed to be clinically insignificant. conc., concentration. AUC_0‐24_; area under the concentration‐time curve from time zero to 24 hours, AUC_60 days_; area under the concentration‐time curve from time zero to 60 days, C_MAX_; maximum concentrations, CL/F; oral clearance, Day 7 conc.; day 7 concentration of piperaquine, F; relative bioavailability, k_a_; absorption rate constant from last transit compartment to central compartment, MTT; mean transit time, Q_P_/F; inter‐compartment clearance, T_MAX_; time to maximum concentrations, t_1/2_; terminal half‐life, V_C/_F; apparent central volume of distribution, V_P_/F; apparent peripheral volume of distribution

Primaquine coadministration did not have a significant impact on the pharmacokinetic properties of DHA or piperaquine when evaluated with a stepwise covariate approach. In the full covariate approach for DHA, the impact of primaquine coadministration was less than ±25% on primary pharmacokinetic parameters (Figure 3A). The full covariate approach for piperaquine resulted in a median 37.3% (95% CI −67.6%, 33.7%) decrease in central volume of distribution and a median 26.8% (95% CI −21.2%, 62.5%) increase in mean transit absorption time during concomitant administration of primaquine (Figure 3B). However, the 95% CI for these covariate effects included a zero effect, so a lack of effect could not be excluded. The impact on other primary pharmacokinetic parameters was less than ±25%. Furthermore, no substantial differences were evident in secondary exposure parameters of DHA and piperaquine in the full covariate approach (Figure 3A and B).

### Electrocardiographic effects of piperaquine

Individually estimated subject–specific QT corrections were slightly less affected by heart rate compared with standard Bazett and Fridericia corrections. Individual regression of QTc and RR intervals resulted in 6/16, 6/16, and 5/16 individuals with regression slopes significantly different from zero using Bazett, Fridericia and individually determined corrections, respectively. Therefore, individual corrections were applied to the observed QT interval. The initial concentration–response analysis showed no significant relationship between piperaquine drug concentrations and ΔQRS (*P* = 0.520). Hence, ΔJTc and ΔQTc showed an almost identical concentration–response relationship (data not shown) and ΔQTc was therefore carried forward in the analysis as this measurement is commonly reported in the literature.

A linear direct response model resulted in an adequate description of the relationship between piperaquine exposure and QTc prolongation. The linear model showed better model fit and predictive performance compared with the other models evaluated (i.e. the power model and E_MAX_ model). The implementation of a delayed response model was not supported by the observed data and resulted in low parameter precisions. The population baseline ΔΔQTc prolongation was estimated close to zero and therefore fixed to this value but allowed for between‐subject variability in the same parameter. No major between‐subject variability was observed in other pharmacodynamic parameters in the final model. Primaquine did not affect the relationship and no other significant covariates (age, gender and electrolyte levels) were identified in the stepwise covariate approach. Within the concentration range measured, the final model resulted in a population mean increase in ΔΔQTc of 4.17 (95% CI 0.973, 43.1) ms with every 100 ng ml^–1^ increase in piperaquine plasma concentration.

The final model showed a satisfactory goodness of fit (Figure 1) and predictive performance, as illustrated by the visual predictive check (Figure 2C and 2D). Eta shrinkage of the slope parameter was moderate (26.5%) and epsilon shrinkage was low (2.39%). A numerical predictive check (*n* = 2000) resulted in 4.50% (95% CI 1.80%, 8.56%) and 4.05% (95% CI 1.80%, 8.56%) of observed ECG measurements below and above, respectively, the simulated 90% prediction interval. Pharmacodynamic parameter estimates from the final model are summarized in Table 2.

**Figure 4:**
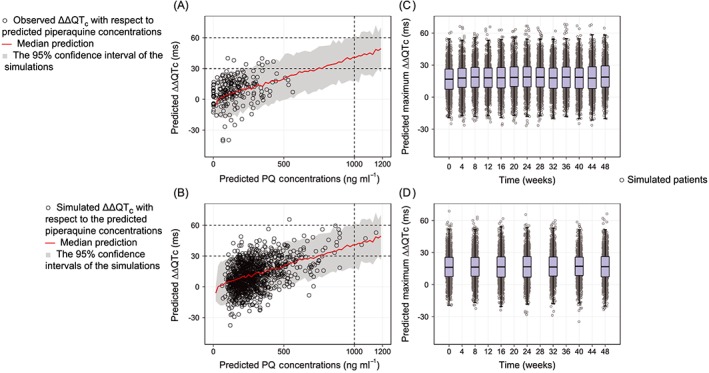
Simulations of QT prolongations in healthy volunteers at different piperaquine (PQ) concentrations (A), after standard 3‐day treatment in patients with uncomplicated *Plasmodium falciparum* malaria (B), after monthly mass drug administration of the standard 3‐day regimen (C), and after bimonthly mass drug administration of the standard 3‐day regimen (D). Box and whisker plot represent the interquartile range and the 2.5^th^ to 97.5^th^ percentiles. ΔΔQTc, double‐delta‐corrected QTc prolongation

Pharmacokinetic–pharmacodynamic model simulations, based on the assumption that a linear concentration–effect relationship continued at piperaquine plasma levels over 500 ng ml^–1^, showed that 95% of all subjects (i.e. 95% prediction interval) had a predicted QT prolongation below 60 ms at piperaquine concentrations below 1000 ng ml^–1^ (Figure 4A). Pharmacokinetic–pharmacodynamic model simulations, using previously published pharmacokinetic parameter estimates in patients with uncomplicated *P*. *falciparum* malaria, resulted in a predicted median QT prolongation of 11.2 (95% CI −15.6, 41.2) ms after standard 3‐day DHA–piperaquine treatment, which is consistent with the current study (Figure 4B). Simulations of monthly and bimonthly mass drug administration regimens over a total duration of 1 year suggest that individually predicted maximum QT prolongations did not reach 50 ms in any subjects [median 18.9 (95% CI −6.44, 49.0) ms after monthly treatment; median 16.8 (95% CI −11.0, 45.1) ms after bimonthly treatment] (Figure 4C and D).

## Discussion

The antimalarial combination treatment of DHA–piperaquine has been used extensively and shown excellent efficacy and tolerability 1, 42. However, recent concerns have been raised regarding the potential for cardiotoxicity because piperaquine, like many drugs in this class, causes delayed ventricular repolarization (manifested as electrocardiograph prolongation of the QT interval). The present study in healthy subjects assessed the pharmacokinetic properties of DHA and piperaquine, potential drug–drug interactions of concomitant primaquine treatment, and QT prolongation associated with piperaquine treatment. The results were generally reassuring, and suggested that it is highly unlikely for marked QT prolongation to occur following standard doses of DHA–piperaquine. Limitations of the study included the small number of participants, the fact that only Thai volunteers were included and that there was a gender bias (three males and 13 female). Thus, modelling and simulation results should not be extrapolated directly to patients with malaria, and especially young children, without considering disease effects, body size differences and enzyme maturation in very young children. Furthermore, the relationship between QT prolongation and the risk of sudden death is not straightforward; the risk of arrhythmia associated with a long QT interval is clearly greater with some drugs than others. Larger population‐based pharmacokinetic–pharmacodynamic studies in patients with malaria and in healthy subjects are needed for final conclusions to be reached on the safety of DHA–piperaquine.

The population pharmacokinetic properties of DHA were best described by a two‐compartment disposition model with six transit compartments in the absorption phase. In previous studies, both one‐ and two‐compartment disposition models have been used to describe the pharmacokinetic properties of DHA 8, 43, 44, 45. The difference in the disposition models reported most likely result from the rapid disposition phase and the different sampling frequencies in the absorption and disposition phases. Sparse sampling is likely to mask an early disposition phase. However, the clinical impact of using a one‐ or two‐compartment structure may well be very small, as long as the terminal elimination half‐life is characterized accurately.

The implementation of body weight as an allometric function on clearance and volume parameters has been reported in previous studies 7, 8. Even though body weight did not provide an improved model fit in the present study, it was retained as a covariate in the final model based on prior biological knowledge and to allow for extrapolation of the developed model into other populations, such as children. No significant covariates were found in the present study, using a step‐wise covariate approach. Modelling performed here demonstrated large variability in the absorption characteristics of DHA – a large between‐occasion variability in mean transit time (52.6%), absorption rate constant (89.0%) and relative bioavailability (35.9%). This might be due to the lipophilic physicochemical properties of DHA, resulting in variable absorption characteristics on different dosing occasions 7. The final model showed a satisfactory goodness of fit and predictive performance (Figure 1 and 2A). Overall, pharmacokinetic parameter estimates were in agreement with those previously reported in healthy volunteers and patients with uncomplicated *P. falciparum* malaria 7, 43, 44, 45, 46.

Piperaquine was described by a three‐compartment disposition model, which is in agreement with recently published studies 8, 47, 48, 49, 50. The variable absorption characteristics of piperaquine were best described with two transit absorption compartments, compared with three or five in previous studies 8, 48. The difference in the number of transit compartments might be explained by different study designs and sample frequencies during the absorption phase.

Body weight, implemented as an allometric function on clearance and volume parameters, improved the model fit. It also has a strong biological prior and has been identified in previous studies 8, 33, 48. No other significant covariates were found in the present study, using a step‐wise covariate approach. The final model showed overall satisfactory goodness‐of‐fit and predictive performance (Figure 1 and 2B). Modelling performed here demonstrated moderate variability in the absorption characteristics of piperaquine (below 35%). The overall pharmacokinetic parameter estimates were in agreement with previous studies in healthy volunteers and patients with *P. falciparum* malaria 8, 23, 48, 50, 51.

The WHO suggested recently that a single low dose of primaquine (0.25 mg kg^–1^) be added to ACTs in order to reduce malaria transmission in low transmission areas 4. The safety of a single low dose of primaquine has been demonstrated in both G6PD‐deficient and G6PD‐normal patients 52, 53 and might be an important tool in malaria elimination efforts 54. To the best of our knowledge, potential pharmacokinetic drug–drug interactions have not been evaluated formally with a modelling approach. This was assessed with two different approaches. First, a bottom‐up approach was performed by characterizing the impact of primaquine coadministration on each pharmacokinetic parameter using a stepwise addition and elimination approach. In the second approach, a top‐down analysis was employed by including a categorical primaquine coadministration effect on all pharmacokinetic parameters simultaneously (i.e. full covariate approach). None of these approaches found any clinically significant drug–drug interactions between primaquine and DHA or piperaquine. However, the full covariate approach indicated a trend of decreasing inter‐compartmental clearance and absorption rate constant of DHA when coadministered with primaquine. Similarly, a trend of decreasing central volume of distribution and increasing mean transit absorption time of piperaquine was seen when coadministered with primaquine. However, the 95% CI of these effects spanned zero, and a lack of effect could not be excluded. A lack of clinically relevant drug–drug interactions with primaquine was further supported by no substantial differences in secondary exposure parameters of DHA and piperaquine, with and without coadministration of primaquine, when using the full covariate approach. These results were expected as primaquine does not induce or inhibit any enzymes and the test compounds are metabolized through different enzymatic pathways 55, 56, 57, 58. The results of the present study were also in agreement with the noncompartmental analysis of the data, which did not identify any drug–drug interactions with primaquine 23.

Many antimalarial drugs have been associated with QT prolongation, which reflects a delay in the repolarization of the ventricular myocytes during the cardiac cycle 16. This can predispose to the development of ventricular arrhythmias, most notably torsade de pointes, and sudden death. Drugs can increase the risk of QT prolongation by several mechanisms, most commonly by blocking the hERG potassium channel and other cardiac ion channels (i.e. carrying calcium and sodium). The antimalarial drug halofantrine was withdrawn from clinical use because it induced marked QT prolongation and was associated with an increased risk of sudden death 59. On the other hand, amiodarone blocks the hERG potassium and calcium/sodium channels, resulting in substantial QT prolongation, but carries a very low risk of degenerating into torsade de pointes 60. The exact relationship between electrophysiological events, QT prolongation and the development of torsade de pointes has not been well characterized. DHA–piperaquine treatment has been associated with QT prolongation both in patients and healthy volunteers but not with torsade de pointes or sudden death 18, 19, 20, 21, 22, 61. Yet, few studies have investigated the relationship between piperaquine exposure and QT prolongation, and no previous studies have quantified this relationship using population pharmacokinetic–pharmacodynamic modelling 20, 62.

No significant QT prolongation has been seen previously with the administration of primaquine 63, 64. The lack of a concentration–response relationship between primaquine concentrations and ΔQTc in the present study confirmed that primaquine, at these doses, has no impact on ventricular repolarization. Although there is some evidence from experimental studies that artemether may prolong the QT interval, the general consensus is that the artemisinin derivatives at currently used doses have no significant effect. Thus, only piperaquine plasma concentrations were used to drive the pharmacodynamic QT prolongation in the present modelling exercise, and the administration of primaquine alone was used as a negative control arm. ΔΔQTc intervals were used in the pharmacodynamic model, to minimize the impact of heart rate and the naturally occurring circadian rhythm of the QT interval 65. This also reduces regression towards the mean of the baseline QT interval, by subtracting the average of the individual baseline values of the QT intervals from the QT measurements. Therefore, a change in the ΔΔQTc interval should be attributed solely to the exposure to piperaquine. In the present study, a significant relationship between QT prolongation and piperaquine concentration was described accurately by a linear exposure–response model, which has also been seen previously 20, 62. Inclusion of electrolytes (potassium and sodium) or any other covariate did not have a significant effect in the model, most likely due to the fact that healthy volunteers were studied here. The final pharmacokinetic–pharmacodynamic model showed overall good diagnostic/predictive performance (Figure 1 and Figure 2C) and the estimated slope was in agreement with that in previous studies 20, 62, indicating that this model was suitable for simulations. There were no significant changes in other electrocardiographic intervals associated with drug administration.

A drug‐induced QT prolongation of less than 60 ms is generally accepted as a clinical cardiac safety stopping rule according to the US Food and Drug Administration (FDA) 66. Simulations, using the final pharmacokinetic–pharmacodynamic model and assuming a continuous linear concentration–effect relationship, predicted that piperaquine concentrations below 1000 ng ml^–1^ would result in a QT prolongation of less than 60 ms in healthy volunteers (i.e. upper end of the 95% CI below 60 ms). A standard 3‐day dosing regimen of 50 mg kg^–1^ in DHA–piperaquine given to pregnant and nonpregnant women with uncomplicated *P*. *falciparum* malaria reported a median maximum piperaquine concentration of 244 ng ml^–1^ (interquartile range 173–344 ng ml^–1^) 8. Thus, standard treatment regimens should result in QT prolongations well below 60 ms and should be safe in a clinical setting. This was further supported by simulations 8, using the developed exposure–response model for QT prolongation. Simulations of standard oral DHA–piperaquine 3‐day treatment in patients with uncomplicated *P. falciparum* malaria resulted in a median QT prolongation of 6.50 (95% CI −18.6, 35.2) ms. The FDA threshold for regulatory concern is 5 ms (the upper limit of the 95% CI being 10 ms) for novel drugs. Even though the QT prolongation of piperaquine shows a somewhat inflated confidence interval, it should not pose a clinical concern at therapeutic concentrations 66.

DHA–piperaquine is a promising candidate for mass drug administration and malaria elimination strategies because of its long terminal elimination half‐life and subsequent long postdose prophylactic effect 9, 67. However, the long half‐life of piperaquine results in accumulation and a 336% (range 271–402%) and 267% (range 146–381%) increase in piperaquine trough concentrations at week 36 compared with week 4 after repeated monthly and bimonthly treatment doses, respectively 67. It is therefore necessary to evaluate long‐term cardiac safety before implementation in clinical settings. Simulations of mass drug administration in 1000 healthy subjects in South‐East Asia receiving standard 3‐day DHA–piperaquine treatment, either monthly or bimonthly, predicted QT prolongations of less than 60 ms in all patients (Figure 4C and D). The simulations predicted a minimal accumulation of QT prolongations over the 12 months, owing to the relatively flat slope of the exposure–response relationship (4.17 ms increase for every 100 ng ml^–1^ increase in piperaquine concentrations). A small difference between the various regimens was noted, with a median QT prolongation with the monthly and bimonthly regimens of 18.9 (95% CI −6.44, 49.0) ms and 16.8 (95% CI −11.0, 45.1) ms, respectively. In summary, simulations performed here with use of the developed pharmacokinetic–pharmacodynamic model suggest that the standard treatment regimen of DHA–piperaquine in patients and mass drug administration over 1 year in healthy subjects are likely to be safe according to standard cardiac safety criteria.

In conclusion, the pharmacokinetic properties of DHA and piperaquine, the influence of concomitant primaquine administration and the relationship between piperaquine exposure and electrocardiographic measurements were successfully characterized using nonlinear mixed‐effects modelling. Concomitant primaquine administration did not affect the pharmacokinetic properties of DHA–piperaquine, supporting the concomitant use of a single low dose of primaquine as a transmission blocking agent in the treatment of malaria. Piperaquine administration resulted in a significant prolongation of the QT interval but the effect was modest and simulations suggest that mass treatments are unlikely to result in dangerous QT prolongation.

## Competing Interests

This study was a part of the Wellcome Trust–Mahidol University–Oxford Tropical Medicine Research Programme supported by the Wellcome Trust of Great Britain. Part of this work was supported by the Bill and Melinda Gates Foundation. The funding bodies did not have any role in the collection, analysis or interpretation of the data, writing of the manuscript, or in the decision to submit the manuscript for publication. All authors have competed the Unified Competing Interest form at http://www.icmje.org/coi_disclosure.pdf (available on request from the corresponding author) and declare no financial relationships with any organization that might have an interest in the submitted work in the previous 3 years; and no other relationships or activities that could appear to have influenced the submitted work.

*We sincerely thank all volunteers for their participation in this study. We also thank the diligent staff from the Hospital of Tropical Disease, Faculty of Tropical Medicine, Mahidol University*.

## Contributors

P.C., T.W. R.H. and J.T. wrote the first draft of the manuscript. B.H., S.P., P.J., N.J.W., N.P.J.D. and J.T. designed the research. B.H., S.P., P.J. and J.T. performed the research. D.B. measured drug concentrations. P.C., T.W. and J.T. analysed the data. All authors read and approved the final version of manuscript.
